# Catalytically active gold clusters with atomic precision for noninvasive early intervention of neurotrauma

**DOI:** 10.1186/s12951-021-01071-4

**Published:** 2021-10-13

**Authors:** Yunguang Zhang, Si Sun, Haile Liu, Qinjuan Ren, Wenting Hao, Qi Xin, Jiangang Xu, Hao Wang, Xiao-Dong Zhang

**Affiliations:** 1 School of Science, Xi’an University of Posts and Telecommunications, Xi’an, 710121 China; 2 Tianjin Key Laboratory of Low Dimensional Materials Physics and Preparing Technology, School of Sciences, Tianjin University, Tianjin, 300350 China; 3 Tianjin Key Laboratory of Brain Science and Neuroengineering, Academy of Medical Engineering and Translational Medicine, Tianjin University, Tianjin, 300072 China

**Keywords:** Gold clusters, Catalytic activity, Traumatic brain injury, Noninvasive administration

## Abstract

**Background:**

Neurotrauma is a worldwide public health problem which can be divided into primary and secondary damge. The primary damge is caused by external forces and triggers the overproduction of peroxides and superoxides, leading to long-lasting secondary damage including oxidative stress, wound infection and immunological reactions. The emerging catalysts have shown great potential in the treatment of brain injury and neurogenic inflammation, but are limited to biosafety issues and delivery efficiency.

**Results:**

Herein, we proposed the noninvasive delivery route to brain trauma by employing highly active gold clusters with enzyme-like activity to achieve the early intervention. The decomposition rate to H_2_O_2_ of the ultrasmall gold clusters is 10 times that of glassy carbon (GC) electrodes, indicating excellent catalytic activity. The gold clusters can relieve the oxidative stress and decrease the excessive O_2_^·−^ and H_2_O_2_ both in vitro and in vivo. Besides, gold clusters can accelerate the wound healing of brain trauma and alleviate inflammation via inhibiting the activation of astrocytes and microglia through noninvasive adminstration. decrease the peroxide and superoxide of brain tissue.

**Conclusions:**

Present work shows noninvasive treatment is a promising route for early intervention of brain trauma.

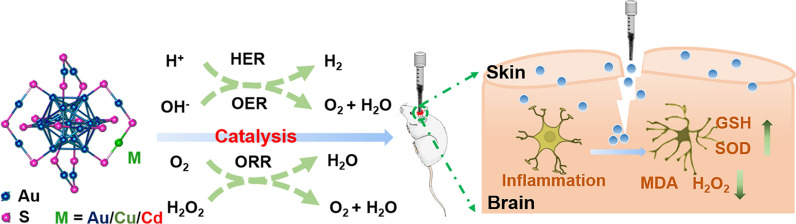

**Supplementary Information:**

The online version contains supplementary material available at 10.1186/s12951-021-01071-4.

## Background

Traumatic brain injury (TBI) has led to globally death and disability with high mobility [1–3] and caused heavily economic burden as a serious public health issue [4–6]. The primary brain damage can trigger a cascade of molecular and biochemical events, leading to long-lasting secondary damage including oxidative stress, wound infection and chronic neurodegenerative diseases [1, 7–9]. Reactive oxygen species (ROS) and reactive nitrogen species (RNS) can oxidatively impair biomacromolecules in vivo and play a crucial role in the pathophysiology of TBI including hydroxyl radical (OH·), superoxide anion radicals (O_2_^·−^), hydrogen peroxide (H_2_O_2_), nitric oxide radicals (NO·) and peroxynitrite (ONOO^−^) [1, 7, 8, 10, 11]. Previous studies have confirmed that TBI can be improved via balancing oxidation reduction by elimination of reactive oxygen and nitrogen species (RONS). Thus, antioxidative biocatalysts with catalase-like (CAT-like), superoxide dismutase-like (SOD-like), peroxidase (POD-like) and glutathione peroxidase-like (GPx-like) activities and good stability show great potential in the treatment and diagnosis of TBI through catalytically scavenging the RONS [12–20].

Earlier studies have shed light on the enzyme-like activities of nanozymes. Carbogenic and gold-based nanozymes exhibited exceeding activity against oxidative stress and neuroinflammation [11, 13, 19, 21, 22]. Nanozymes composed of metals, metallic oxide and alloys like Pt, Pd, Cr, V and Ce possess high enzymatic activities, beneficial for TBI treatment [12, 14, 15, 18, 23, 24]. Especially, the ceria nanozymes showed excellent SOD-like and CAT-like catalytic activities on scavenging RONS via redox cycle between Ce^4+^ and Ce^3+^ [12, 18, 25]. Compared with traditional nanozymes, the gold clusters show controllable modulation in catalytic activity and selectivity, which can be improved by increasing the atomic utilization efficiency at atomic levels as well as atomic engineering via atom manipulation [26]. Unlike traditional nanozyme, bioactive gold clusters exhibit the high efficient renal clearance and negligible toxicity even at very high injected dose (500 mg/kg) [24, 25, 27]. Moreover, due to ultrasmall size, the gold clusters can enter cell or injury site easily, resulting in high efficient treatment [26, 28]. Gold clusters possess activities like SOD, CAT and GPx enzymes, performing significant antioxidant effects in TBI treatment [26, 28–31]. Though nanotechnologies for TBI diagnosis and monitoring were employed to promote the activity and utilization of nanozymatic biocatalysts like atom engineering, surface modification, and size modulation [32–34], it remains painless and chanllenged for clinical translation [12, 15, 29–31, 35–45]. Different from the intravenous injection, the noninvasive diagnosis and stimulation for TBI can achieve rapid and painless treatment, worthy of considering to promote recovery and minimize disability. However, the noninvasive methods for TBI treatment are still highly challenged.

Herein, we investigated a noninvasive administration to treat brain trauma at the early stage with gold clusters of well-defined structure and high catalytic selectively. Electrochemical assay unraveled their high catalytic activities toward hydrogen evolution reaction (HER) and oxygen evolution reaction (OER), and further revealed their excellent activity toward the reduction of O_2_ and H_2_O_2_. Furthermore, noninvasive administration of the gold clusters distinctly improved the wound healing on TBI mice and Morris water maze tests further confirmed significant recovery on learning ability and spatial memory with Au_24_Cu_1_ and Au_24_Cd_1_ treatment. *Ex vivo* assay further proved the ability of gold clusters on mitigating oxidative stress and inhibiting neuroinflammation. Together with the results of acceptable biocompatibility, gold clusters showed promising potential as noninvasive therapeutics against TBI.

## Results and discussion

**Fig. 1:**
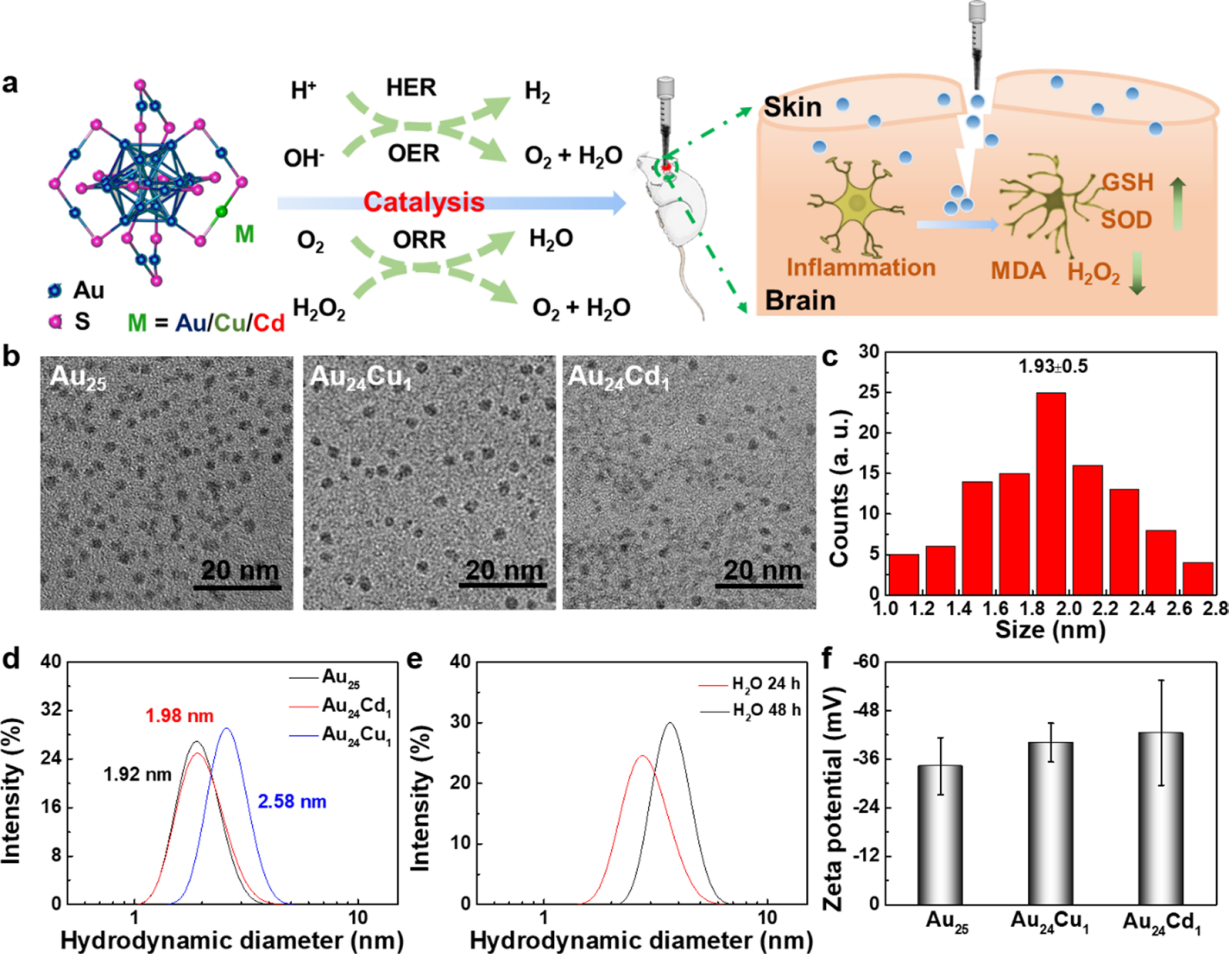
Structural characterization of Au_25_ clusters. **a** Schematic illustration of noninvasive TBI treatment with gold clusters *via* a catalytic system. **b** TEM image of MPA-protected Au_25_, Au_24_Cu_1_ and Au_24_Cd_1_ clusters. Scale bar: 20 nm. **c** Size distribution of MPA-protected Au_25_ nanocluster measured by analyzing 100 nanodots from TEM images. **d** Hydrodynamic diameters of MPA-protected Au_25_, Au_24_Cu_1_ and Au_24_Cd_1_ in PBS buffer, and the mean sizes of Au_25_, Au_24_Cu_1_ and Au_24_Cd_1_ were 1.98, 1.92 and 2.58 nm respectively. **e** Stability of Au_25_ nanocluster in H_2_O after 24 and 48 h at room temperature, and the slightly increase of hydrodynamic diameter revealed satisfying stability. Additionally, the zeta potentials of the clusters **f** were around -35 mV, indicative of good colloid stability (n=3 independent experiments, data are presented as mean ± SD)

The Au_25_ cluster is composed of 13 gold atoms as the core and 6 Au_2_(SG)_3_ as the outer shell. The catalytically active sites of the cluster are mainly located on the surface. Single atom Cu or Cd replaces the Au in one of the S-Au-S, indicative of synthesizing Au_24_Cu_1_ and Au_24_Cd_1_, respectively. After single atom Cu and Cd substitution, the valence electron structure of the cluster is altered, thereby changing the catalytic performance of the cluster [23]. Figure 1a illustrated the effects of MPA-protected Au_25_, Au_24_Cu_1_ and Au_24_Cd_1_ clusters in brain trauma via catalytic systems. TEM images illustrated the homogenous distribution of the clusters at about 2 nm (Fig. 1b, c), similar with L-NIBC-coated gold clusters [46]. The hydrodynamic sizes determined by dynamic light scattering (DLS) were 1.98, 1.92 and 2.58 nm for Au_25_, Au_24_Cd_1_ and Au_24_Cu_1_, respectively, a little larger than the statistical diameter of TEM. After incubation in water for 24 and 48 h, the size of clusters changed negligibly, revealing the favorable stability (Fig. 1d, e). In addition, the zeta potentials of all clusters were around − 35 mV (Fig. 1f), consistent with our previous work [26, 47]. The concentration Cd and Cu elements in Au_24_Cd_1_ and Au_24_Cu_1_ clusters account for 3% and 5% of the total metal as determined by an accurate inductively coupled plasma mass spectrometry (ICP-MS), respectively, further confirming the single-atom substitution in Au_25_ clusters (Additional file 1: Fig. S1). X-ray photoelectron spectroscopy (XPS) confirmed that the dominant state of Au in the clusters is the Au (0) state, and the peaks of Cu 2p_3/2_ (931.8 eV) and Cd 3d_5/2_ (404.8 eV) are on the reducing side of Cu (0) (id. =932.2 eV) and Cd (0) (id. =405.3 eV), respectively, indicating the successful single-atom substitution in Au_25_ clusters (Additional file 1: Fig. S2). These results display the ultrasmall size and good colloid stability of clusters, manifesting the potential in biological applications.

**Fig. 2:**
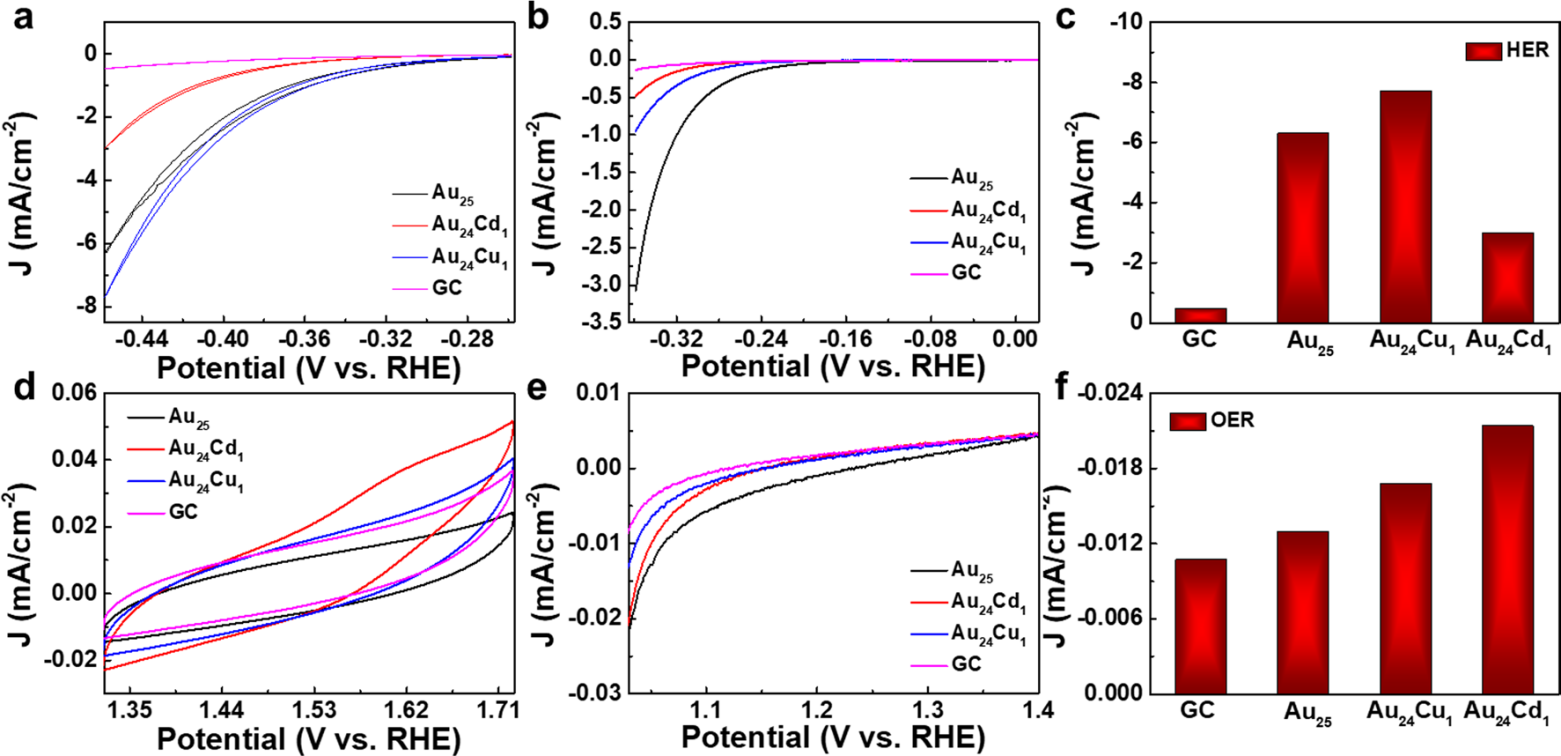
Catalytic performance for overall water splitting. **a** CVs of GC electrode modified with as-prepared Au_25_, Au_24_Cu_1_ and Au_24_Cd_1_ clusters in 0.5 M H_2_SO_4_. Scanning rate: 0.010 V s^−1^. **b** Linear sweep HER voltammograms for Au_25_, Au_24_Cu_1_ and Au_24_Cd_1_ clusters. Scanning rate: 0.001 V s^−1^. **c** Current density of different clusters at the potential of −0.4585 V toward the activity of HER. **d** CVs of GC electrode modified with as-prepared Au_25_, Au_24_Cu_1_ and Au_24_Cd_1_ clusters in 1 M KOH. Scanning rate: 0.10 V s^−1^. **e** Linear sweep OER voltammograms for Au_25_, Au_24_Cu_1_ and Au_24_Cd_1_ clusters. Scanning rate: 0.005 V s^−1^. **f** Current density of different clusters at the potential of 1.3244 V toward the activity of OER

To evaluate the electrocatalytic activities of the Au_25_, Au_24_Cu_1_ and Au_24_Cd_1_ clusters, a standard three-electrode system were employed to conduct the electrocatalytic activities toward HER and OER [48–50]. Figure 2a shows cyclic voltammetry (CV) curves of glassy carbon (GC) electrodes modified with as prepared Au_25_, Au_24_Cu_1_ and Au_24_Cd_1_ clusters. Compared with a blank GC electrode, clusters achieve larger negative current density toward HER (Fig. 2a), which were further verified by linear sweep voltammetry (LSV) measurement (Fig. 2b). As shown in Fig. 2c, Au_24_Cu_1_ clusters demonstrated a higher efficiency at the potential of −0.4585 V in catalysis, significantly better than Au_25_(SC_12_H_25_)_18_ [8] and PtCo cluster [10]. OER exhibits similar results with HER that all clusters improved the performance of the electrochemical reaction (Fig. 2d, f). Au_24_Cd_1_ shows the lowest current onset on CV curves at 1.3 V, followed by Au_24_Cu_1_ and Au_25_, which is a little different from the results of HER. Besides, Au_24_Cd_1_ can reach 0.023 mA/cm^2^ at a potential of 1.3254 V, while that of NiFe clusters is less than 0.01 mA/cm^2^, indicating better superiority to NiFe cluster [11]. The results of HER and OER further demonstrated that the catalytic performance were altered by the valence electron structure change via different single-atom substitution [23].

**Fig. 3:**
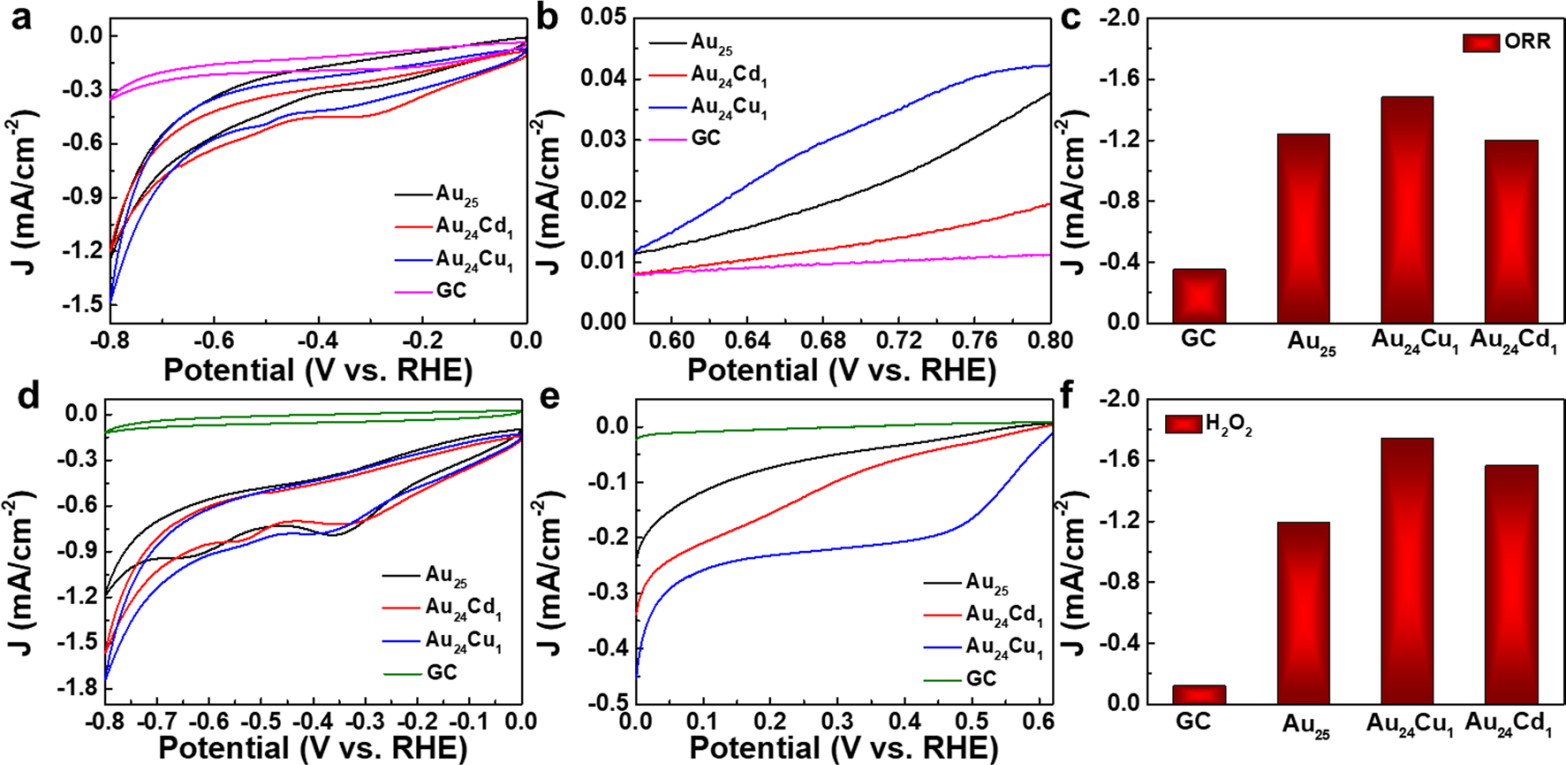
Catalytic activities of the Au_25_ clusters. **a** CVs of GC electrode modified with as-prepared Au_25_, Au_24_Cu_1_ and Au_24_Cd_1_ clusters in O_2_-saturated 0.01 M PBS (pH 7.4). Scanning rate: 0.050 V s^−1^. **b** LSV of GC electrode modified with as-prepared Au_25_, Au_24_Cu_1_ and Au_24_Cd_1_ clusters in O_2_-saturated 0.01 M PBS (pH 7.4). Scanning rate: 0.005 V s^−1^. **c** Compared the ORR activities of Au_25_, Au_24_Cu_1_ and Au_24_Cd_1_ clusters at the potential of −0.8 V. **d** CVs of GC electrode modified with as-prepared Au_25_, Au_24_Cu_1_ and Au_24_Cd_1_ clusters in the presence of 9.8 mM H_2_O_2_ in N_2_-saturated 0.01 M PBS (pH 7.4). Scanning rate: 0.050 V s^−1^. **e** LSV of GC electrode modified with as-prepared Au_25_, Au_24_Cu_1_ and Au_24_Cd_1_ clusters in the presence of 9.8 mM H_2_O_2_ in N_2_-saturated 0.01 M PBS (pH 7.4). Scanning rate: 0.010 V s^−1^. Rotation rate: 1600 rpm. **f** Compared the H_2_O_2_ scavenging activities of clusters at the potential of -0.8 V

We also evaluated the in vitro catalytic activities of Au_25_, Au_24_Cu_1_ and Au_24_Cd_1_ clusters in H_2_O_2_ reduction and oxygen reduction reaction (ORR) [51, 52]. As shown in Fig. 3a, the current density of the GC electrode modified by Au_24_Cu_1_ clusters can reach -1.48 mA/cm^−2^ at a potential of -0.8 V, a little higher than Pt_12_ clusters [18]. The results were further verified by LSV measurement (Fig. 3b), demonstrating that all clusters can enhance the electrocatalytic activity and Au_24_Cu_1_ clusters exhibit the best activity for ORR among all clusters. For the reduction of H_2_O_2_, only imperceptible reduction current was observed in the presence of H_2_O_2_ on the GC electrode, consistent with previous works [51]. Compared with the GC electrode at -0.8 V, Au_24_Cu_1_ clusters show the current destiny of -1.74 mA/cm^−2^, and Au_24_Cd_1_ clusters exhibit that of -1.56 mA/cm^−2^ (Fig. 3d,  e), slightly better than noble metal (Ag, Pd, Au, Pt) on Graphene/ZnO multihybrid nanoarchitectures [19]. Figure 3c, f quantitatively demonstrate the improvement of all clusters on O_2_ and H_2_O_2_ reduction, indicating excellent catalytic activities. Au_24_Cu_1_ presented the predominance toward both reactions, and Au_24_Cd_1_ and Au_25_ followed toward H_2_O_2_ and O_2_ reduction, respectively.

**Fig. 4:**
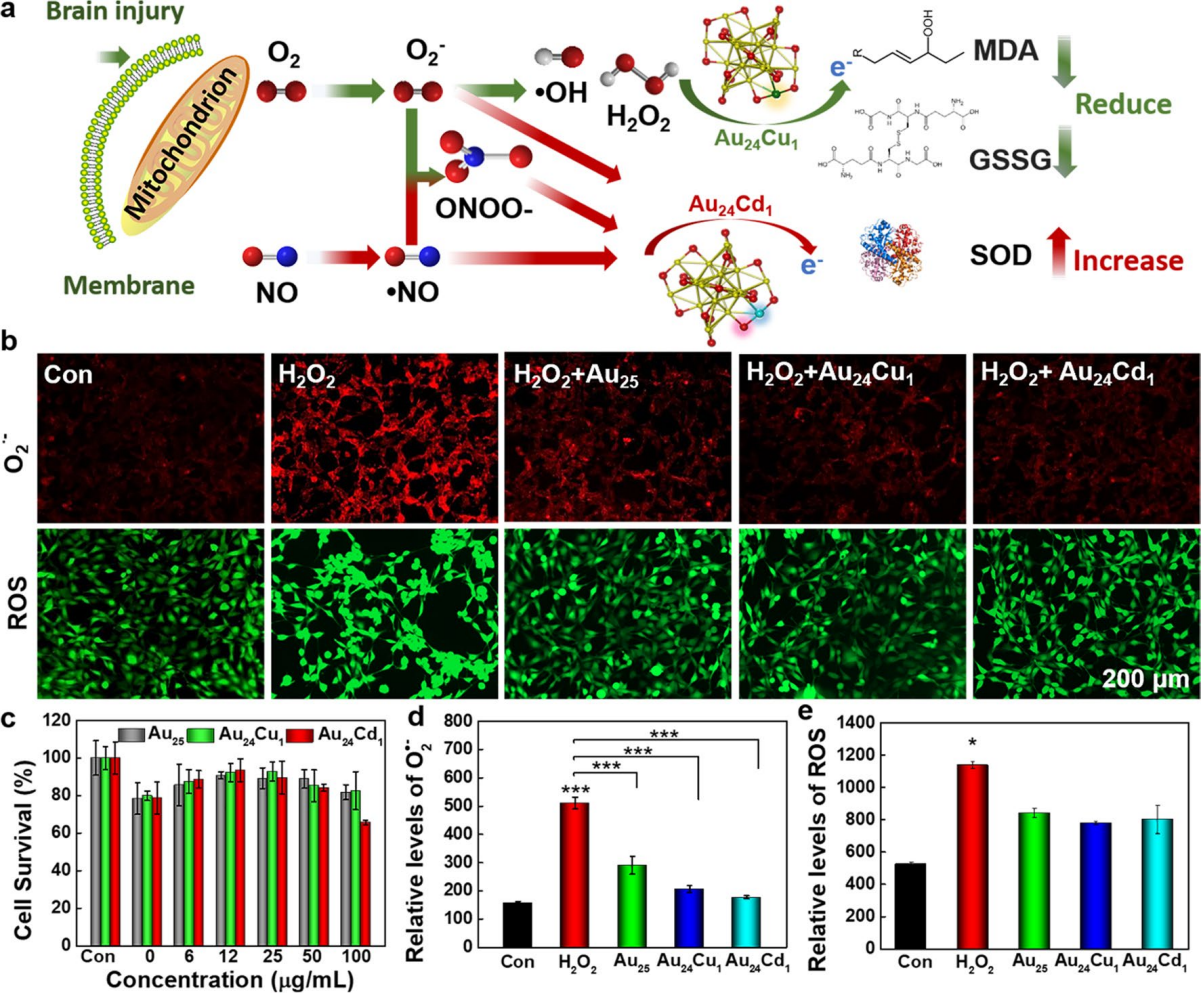
Catalytic mechanism and *in vitro* evaluation of the clusters. **a** Schematic illustration of the catalytic mechanism and selectivity of the clusters. Cu and Cd as single active sites exhibit superiorities against reactive oxygen species (ROS) and reactive nitrogen species (RNS) respectively, resulting in free radical scavenging and inspire their use in brain injury. **b** Fluorescence images of ROS (green) and O_2_^·−^ (red) levels induced by 100 µM H_2_O_2_ with or without clusters treatment, illustrating the high activity of scavenging ROS of the clusters. **c** HT22 cell viability under treatment of H_2_O_2_ and treated with or without the clusters (n = 5 per group, data are presented as mean ± SD), which showed the ability of the clusters to rescue the lethality induce by H_2_O_2_. **d**, **e** Quantitative analysis of Au_25_, Au_24_Cu_1_ and Au_24_Cd_1_ against O_2_^·−^ and ROS, respectively (n = 3 independent experiments, data are presented as mean ± SD). Data are analyzed by one-way ANOVA with Tukey test (adjusted p values are shown, *p< 0.05, ***p< 0.001, compared with the Con group)

Since the remarkable catalytic activities inspired us to investigate their biological responses in cells and brain-injured mice, we conducted biological inspections to evaluate their activities in vitro and in vivo. Combined with our previous work [26], Au_24_Cd_1_ and Au_24_Cu_1_ clusters conferred catalytic selectivity and enzyme-like activities, beneficial to reduce the oxidative stress induced by brain injury (Fig. 4a). Au_25_, Au_24_Cu_1_ and Au_24_Cd_1_ exihibit good biocompatibility at the concentration of 50 µg/mL to different neural cell lines (HT22, BV2 and MA-c) determined by 3-[4,5-dimethylthiazol-2-yl]-2,5 diphenyltetrazolium bromide (MTT) assays (Additional file 1: Fig. S3), presenting favorable metabolic properties [26].

To investigate the biological activity of clusters, imaging of H_2_O_2_-treated neuron cells with or without Au_25_, Au_24_Cd_1_ and Au_24_Cu_1_ clusters was obtained (Fig. 4b). The H_2_O_2_ stimulation can significantly elevates signals, indicating the presence of excessive amount of ROS and O_2_^·−^ [53]. All clusters can decrease the ROS and O_2_^·^^−^ signals, while Au_24_Cd_1_ shows the best clearance efficiency. Meanwhile, Au_24_Cu_1_ displays better clearance capability against ROS than Au_25_ and Au_24_Cd_1_, suggesting higher selectivity for ROS. The related quantification further confirmed the remarkable biological catalytic activity and laying the groundwork for *in vivo* utilization (Fig. 4d, e). In addition, H_2_O_2_ induces decreases in cell viability (∼78%) due to oxidative stress and inflammation [54], while Au_25_, Au_24_Cd_1_ and Au_24_Cu_1_ clusters can rescue cell viability back to 90%, indicative of great potential to protect nerves and lower the lethality from H_2_O_2_-induced cytotoxicity (Fig. 4c). All the in vitro results manifest good catalytic activities of Au_24_Cd_1_ and Au_24_Cu_1_ clusters on ROS scavenging, revealing their potential as biocatalysts and suggesting further investigation in vivo.

**Fig. 5:**
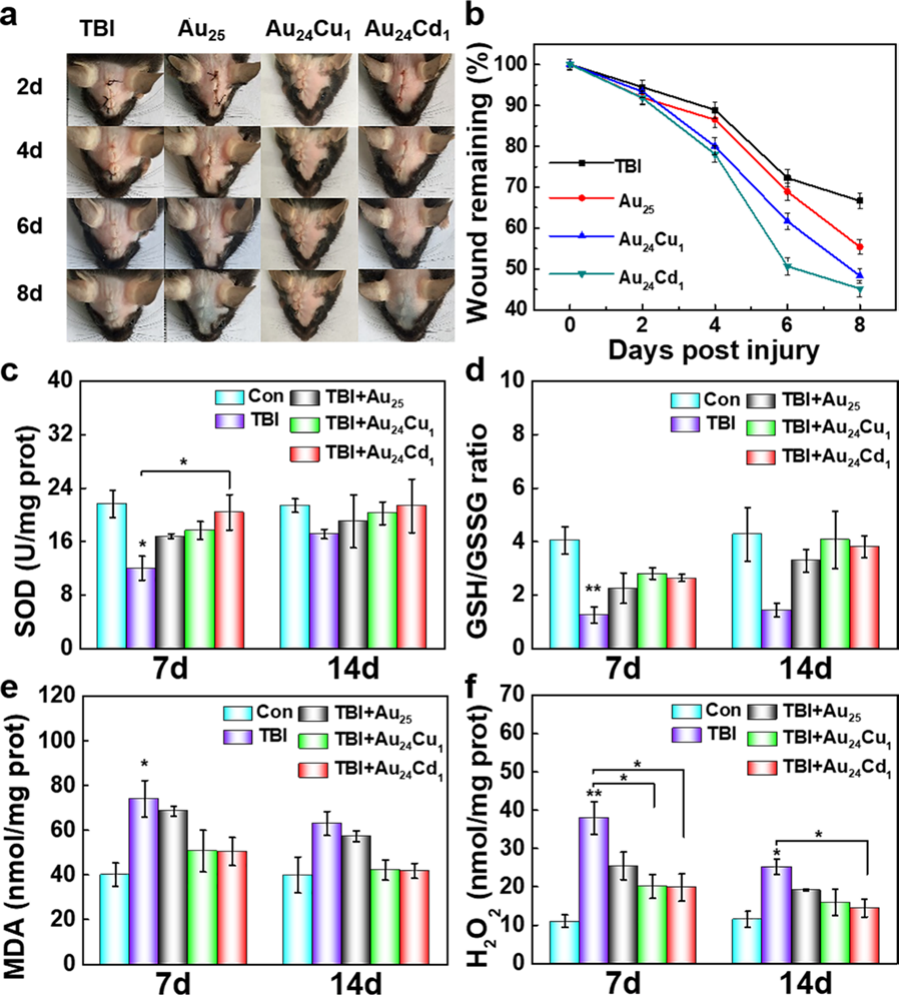
In vivo investigation of the clusters. **a** Wound healing processes over time of TBI mice with and without treatment of clusters, and **b** showed wound remaining percentage over time. The Au_24_Cu_1_ and Au_24_Cd_1_ showed favorable therapeutic effect against TBI. **c**–**f** Indicators for oxidative stress, including SOD, GSH/GSSG, MDA, and H_2_O_2_, of TBI mice with or without treatment of clusters on days 7 and 14 post intervention (n = 3 per group). The clusters can rescue the level of SOD and GSH/GSSG decreased by TBI, and decrease the MDA and H_2_O_2_, indicative of alleviating the oxidative stress *in vivo*. Data are presented as mean ± SEM and compared with the Con groups, analyzed by one-way ANOVA with Tukey test (adjusted p values are shown, *p< 0.05, **p< 0.005)

The primary injuries triggers a casade of biochemical reactions, leading to the long-lasting sencondary injuries. The secondary brain injuries generate harmful molecules and cytokines, leading to acute or chronic neuronal damage, memory impairment and inflammation. Traditional intravenous administration have shown great poteintial in brain diseases, but toxicity remains a major concern for clinical translation [15, 28]. Therefore, the above *in vitro* preliminary results inspired us to treat TBI noninvasively and it is reasonable to develop a noninvasive routine to treat TBI at the early stage. The clusters were dropped in the injured area of TBI mice. The wound size was significantly reduced to healthy levels after clusters treatment, whereas the untreated mice only showed a partial recovery (Fig. 5a, b). We further evaluated the oxidative stress-related indicators, including SOD, GSH/GSSG, MDA, and H_2_O_2_ in TBI mice [28, 55]. Brain injuries generate excessive ROS in tissues, consume lots of SOD and GSH, and produce harmful lipid peroxidation (Fig. 5c–f). SOD and GSH/GSSG are sharply decreased on day 7 post-injury and slightly increased on day 14 post-injury, and MDA and H_2_O_2_ are relatively serious on day 7 post-injury and a little alleviated on day 14 post-injury for TBI groups, indicating severe oxidative stress after brain injuries. However, clusters can effectively decrease the MDA and H_2_O_2_ levels, suggesting the ROS elimination of clusters, and can siginificantly recovered the SOD and GSH/GSSG levels, maintaining the balance of ROS levels.

**Fig. 6:**
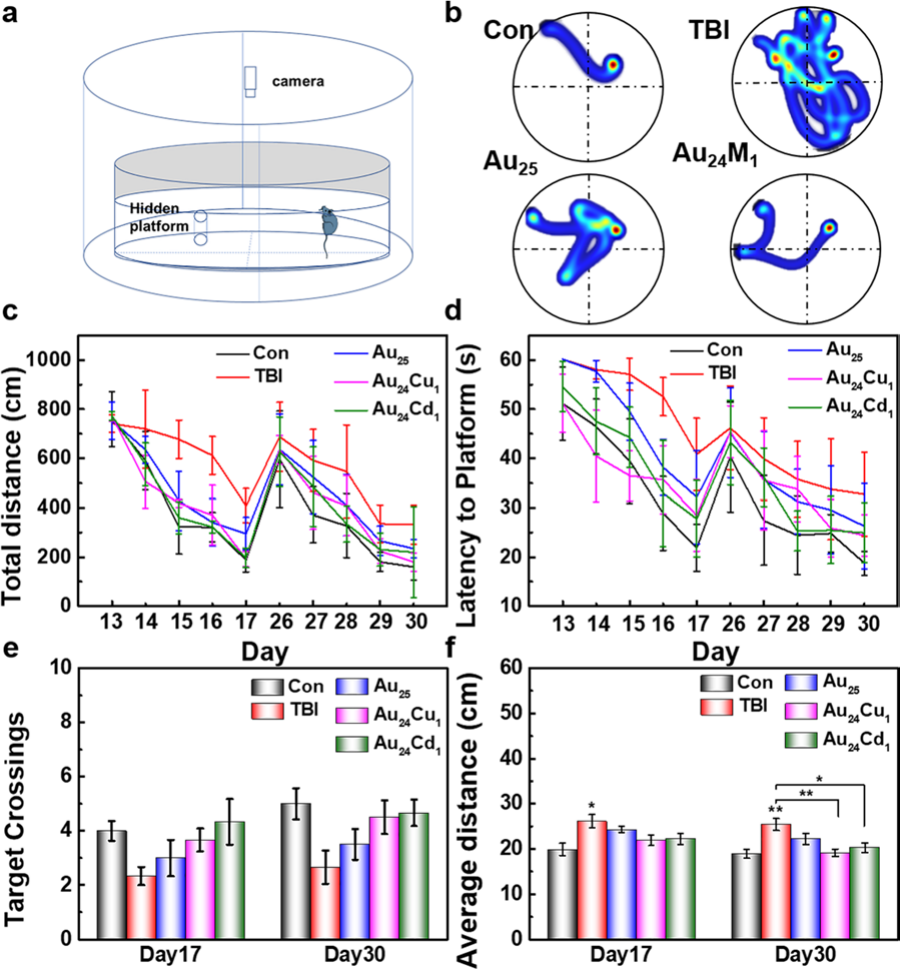
Morris water maze tests. **a** Schematic illustration of the apparatus. **b** Paths of tested mice to the platform treated with or without the clusters. **c** Distance traveled to the hidden platform, and **d** latency to locate and rest on the hidden platform of the tested mice on days 13–17 and 26–30. The number of platform crossings (**e**) and average distance to locate the platform of the tested mice recorded on day 17 and 30. Results illustrated that the clusters especially the Au_24_Cu_1_ and Au_24_Cd_1_ promoting the recovery of neuronal cognition and the spatial learning and memory abilities of TBI mice. Data are presented as mean ± SEM and compared with the Con groups, analyzed by one-way ANOVA with Tukey test (adjusted p values are shown, *p< 0.05, **p< 0.005)

Moreover, the behavior tests were conducted to evaluate the spatial learning and memory abilities by Morris water maze (Fig. 6). All mice were trained to learn to search for the platform during the acquisition phase on days 13–17 and 26–30 including the total distance travelled and the latency to the hidden platform. Compared with TBI groups, both travel distance and latency to platform gradually decrease with training after clusters treatment (Fig. 6c, d), indicating that clusters can effectively improve the motor function following brain injury. Au_24_Cu_1_ or Au_24_Cd_1_ clusters show better results than Au_25_, indicating the efficiency enhancement by Cu and Cd single-atom substitution. Figure 6e, f show that platform crossings and average distance obviously decrease in TBI group and can almost return back to normal levels with cluters treatment after injury. These facts imply that clusters can effectively improve the learning ability and spatial memory of mice with TBI-induced brain injury.

**Fig. 7:**
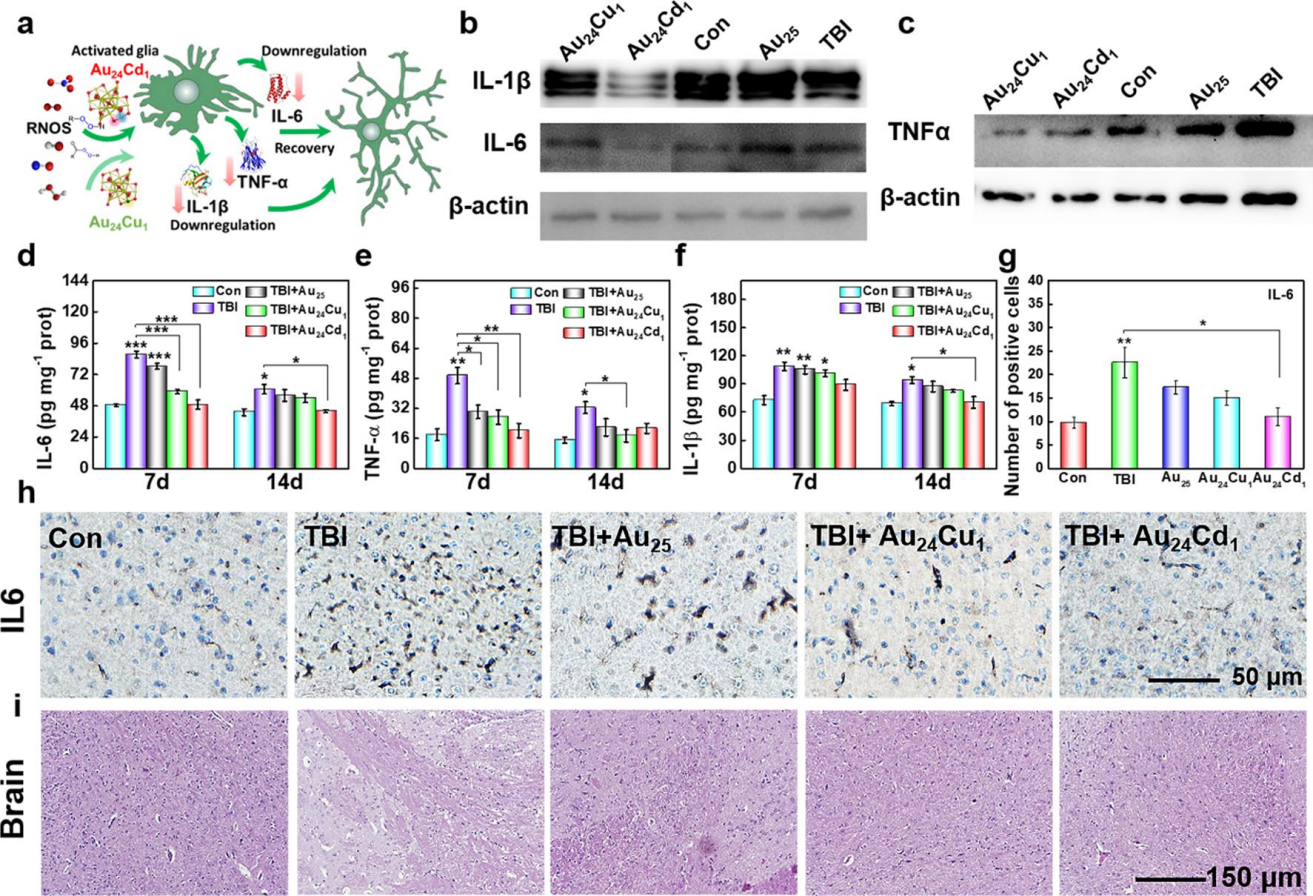
Inflammation levels in brain tissues. **a** Schematic illustration of catalytic activity on oxidative stress and inflammatory responses of the clusters. Expression levels of IL-1β, IL-6 (**b**), and TNF-α (**c**) in the brain tissues on day 30 post TBI with or without clusters (n =3 per group) were analyzed using western blotting. The Au_24_Cd_1_ showed superiority on downregulating the IL-1β and IL-6, while the Au_24_Cu_1_ had better effect toward TNF-α. **d**–**f** ELISA analysis of inflammatory factors on days 7 and 14 post TBI with or without treatment of clusters (n = 3 per group) further confirmed the abilities of the Au_24_Cu_1_ and Au_24_Cd_1_ on regulating inflammatory factors. All the samples were derived from the same experiment and blots were processed in parallel. Data are presented as mean ± SEM and compared with the control groups. **g** Quantitative analysis of the positive cell numbers in injured cortex with or without nanocluster treatment (n=3 per group). Analyzed by one-way ANOVA with Tukey test and compared with the Con groups. **h** The immunohistochemical assay illustrated the conspicuous effect of Au_24_Cd_1_ and Au_24_Cu_1_ against IL-6. **i** The pathological slices showed that the TBI group exhibiting apoptotic and swollen nerve cells and infiltration of inflammatory cells, while the groups treated with clusters especially the Au_24_Cd_1_ and Au_24_Cu_1_ recovered to almost normal. Data are presented as mean ± SEM and compared with the Con groups, analyzed by one-way ANOVA with Tukey test (adjusted p values are shown, *p< 0.05, **p< 0.005, ***p< 0.001)

Since brain injuries can lead to chronic inflammation, we were examined their therapeutic effects on the neuroinflammation. Figure 7a demonstrates the schematic illustration for the TBI-associated inflammatory responses and relevant modulation with the clusters [1, 8]. IL-1β, IL-6 and TNF-α are all upregulated after injury, revealing strong local inflammation. Au_25_ only shows minor downregulation toward the three inflammatory factors, while Au_24_Cd_1_ and Au_24_Cu_1_ exhibit exceptional efficiency to reduce their levels, manifesting that substitution of Cu and Cd atom has great influence on the catalytic activity (Fig. 7b, c). Au_24_Cu_1_ performs better than Au_24_Cd_1_ in downregulating overexpressed TNF-α, whereas Au_24_Cd_1_ behaves better in mediating IL-1β, IL-6, implying catalytic selectivity to different inflammatory cytokines. ELISA kits further verified the immunoblot results that Au_24_Cd_1_ and Au_24_Cu_1_ are superior to Au_25_ on inhibiting inflammatory factors in brain tissues including IL-1β, IL-6, and TNF-α (Fig. 7d, f). Additionally, the immuno histochemical assay illustrated the conspicuous anti-inflammation effect of Au_24_Cd_1_ and Au_24_Cu_1_ against IL-6 (Fig. 7 h), consistent with quantitative results in Fig. 7 g. Furthermore, significant differences on the pathological slices can be seen that the TBI group exhibited swelling of nerve cells and obvious infiltration of inflammatory cells; while the groups treated with clusters especially the Au_24_Cd_1_ and Au_24_Cu_1_ recovered to almost normal (Fig. 7i). These results demonstrated that the clusters especially Au_24_Cd_1_ and Au_24_Cu_1_ can accelerate the wound healing of traumatically injured brain by inhibiting the inflammatory factors.

**Fig. 8:**
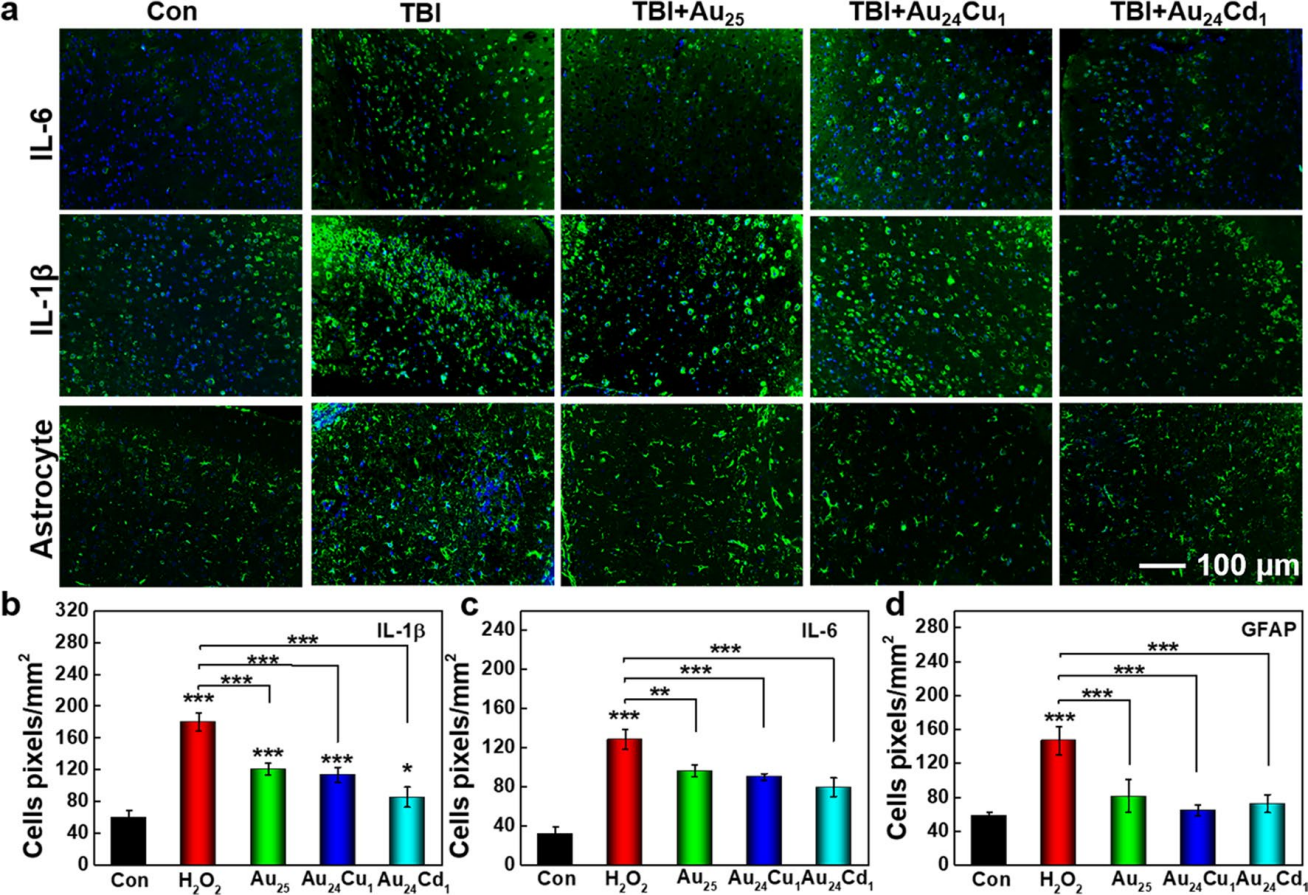
Staining characterization of inflammation levels. **a** Immunofluorescence staining of IL-6, IL-1β and astrocytes (GFAP) in injured cortex 30 days post TBI with or without nanocluster treatment. **b**–**d** Quantitative analysis of the expression IL-6, IL-1β and GFAP with positive cells in the injured cortex with or without nanocluster treatment (n=3 per group). The Au_24_Cd_1_ and Au_24_Cu_1_ presented appealing effects of inhibiting the activation of astrocyte and the expression of IL-6 and IL-1β, which revealed excellent capability of the Au_24_Cd_1_ and Au_24_Cu_1_ on anti-neuroinflammation and further verified the effect of the clusters against TBI. Data are presented as mean ± SEM and compared with the Con groups, analyzed by one-way ANOVA with Tukey test (adjusted p values are shown, *p< 0.05, **p< 0.005, ***p< 0.001)

Brain injuries can also mediate the recruitment of microglia and astrocytes in the injuried areas, which are the key cellular mediators of TBI after brain injury [1]. As shown in Fig. 8a, lots of astrocytes are produced and activated along with inflammatory cytokines, indicating strong local inflammation. The Au_24_Cd_1_ and Au_24_Cu_1_ clusters can inhibit the activation of astrocytes and remarkably reduce the overexpression of IL-1β and IL-6. The relative quantification further exhibited the significant effect of Au_24_Cd_1_ and Au_24_Cu_1_ clusters (Fig. 8b–d). Immune response associated IL-1β and IL-6 can be alleviated by Au_24_Cd_1_, while Au_24_Cu_1_ shows better effect on reducing TNF-α, indicating their catalytic selectivity and potential selectivity towards the treatment of neuroinflammation. Also, it is noted that the performance of mice in Au_24_Cu_1_ and Au_24_Cd_1_ groups is superior to that in Au_25_ groups, indicating that Au_24_Cu_1_ and Au_24_Cd_1_ clusters possess a better anti-inflammation property by efficiently eliminating excessive free radicals and inhibiting neuroglia activation. Additionally, hematology analysis and tissue toxicology analysis showed no toxicity in the long term, suggesting the good biological safety of clusters (Additional file 1: Figs. S4–S6).

The present work demonstrated the high catalytic activity of Au_24_Cd_1_ and Au_24_Cu_1_ clusters and their availability as TBI therapeutics. Unlike traditional nanozymes, gold clusters overcomed the water insolubility and exhibited the well-defined structure at the atomic level with ultrasmall size and good biocompatibility. Catalytic activity and selectivity of clusters can be further increased and availability as TBI therapeutics can also be improved via single ation substitution. Moreover, since noninvasive administration can directly and rapidly release the biocatalysts into the damaged area, it shows promissing potential for TBI treatment, eliminating the RNOS and inhibiting subsequent neuroinflammation [15, 26, 56, 57]. Necessarily, exploiting new biocatalysts with high catalytic activities and favorable biocompatibility, and developing new delivery modes for the biocatalysts for the noninvasive intervention of TBI remain constant challenges in this field [15, 43]. As more biocatalysts with distinguished catalytic activities against oxidative stress are under development [20, 58], there will be more potential therapeutics in the use of noninvasive intervention against TBI.

## Conclusions

In summary, we have reported a noninvasive therapeutic to treat brain trauma at the early stage with gold clusters of well-defined structure and high catalytic selectively. Compared with reported nanozymes, the gold clusters showed higher catalytic activities toward water splitting and reduction of O_2_ and H_2_O_2_ by introducing the Cu or Cd catalytic active site. The biological results reveal that clusters can modulate the oxidative stress and further alleviate neuroinflammation by noninvasive adminstration. Besides, these results conclude that Au_24_Cd_1_ preferentially decreases IL-1β and IL-6, while Au_24_Cu_1_ shows the tendency to decrease TNF-α, indicating their different selectivity for alleviating neuroinflammation. Behaviors and histology illustrated their favorable effects against TBI after noninvasive administration. In conclusion, our work shows the great potential of noninvasive early intervention for the treatment of TBI with enzymatic biocatalysts.

## Materials and methods

### Materials and reagents

All chemicals and reagents were purchased from commercial sources and used without further purification. Gold chloride (HAuCl_4_·3H_2_O) was purchased from Sigma-Aldrich. Copper nitrate (Cu(NO_3_)_2_), Cadmium nitrate (Cd(NO_3_)_2_), 3-Mercaptopropionic acid (MPA), Sodium borohydride (NaBH_4_), Sodium hydroxide (NaOH), were purchased from Aladdin. Ultrapure water (18.2 MΩ*cm) was used for all the experiments. Kits and fluorescent probes were purchased from commercial sources and used as per the instructions.

### Materials preparation, characterization and catalytic activities

The MPA-protected Au_25_, Au_24_Cu_1_ and Au_24_Cd_1_ clusters were prepared as per the previous reports [26, 59, 60]. Briefly, HAuCl_4_ (aqueous, 20 mM, 0.25 mL) and MPA (aqueous, 5 mM, 2 mL) were added to water (2.35 mL) and stirred for 5 min at room temperature. Then, NaOH solution (aqueous, 1 M, 0.3 mL) was added to the reaction mixture, followed by the addition of 0.1 mL of NaBH_4_ (43 mg of NaBH_4_ powder in 10 mL of 0.2 M NaOH solution). Au_25_MPA_18_ was collected after the final reaction mixture stirred at room temperature for 3 h in the dark and aged at 4 °C for 12 h. The Au_24_Cu_1_ and Au_24_Cd_1_ were synthesized based on the same method, except the Au atoms in HAuCl_4_ (20 mM, 0.25 mL) were replaced by various nitrate metal ions (Cu^2+^, Cd^2+^) at a 4% molar ratio. Ultrafiltration tubes of 3 and 10 K at 3500 rpm/min were used for ultrafiltration to remove smaller organic ligands and larger-sized clusters, and lyophilization was used to collect the purified product for further test and investigation.

A JEM-2100 F electron microscope (JEOL, Japan) was employed to acquire transmission electron microscopic (TEM) images. A Malvern Zetasizer Nano ZS90 (UK) was employed to measure dynamic light scattering (DLS) to test the hydrodynamic size and determine the zeta potential of clusters.

The XPS spectrum of the metal elements was performed using ESCALAB Xi+ spectrometer, with a monochromatic Al Kα X-ray source (Thermo Fisher Scientific). All XPS spectra are processed with XPSPEAK41 software and corrected with the peak of C1s (standard: 428.8 eV). ICP-MS (Agilent, US) was used to determine the content of each metal element in the clusters.

The clusters were deposited onto the surface of glassy carbon (GC) electrodes for electrochemical assay [14, 40]. Briefly, as-prepared MPA-protected Au_25_, Au_24_Cu_1_ and Au_24_Cd_1_ clusters (20 µL, 0.5 mg/mL) were deposited onto the surface of GC electrodes and dried naturally, then Nafion solution (3 µL) was dropped onto the surface of GC electrodes and dried, the modified electrodes were used for catalytic activity tests.

Cyclic Voltammetry (CV, scan rate: HER, 10 mV s^−1^; OER, 100 mV s^−1^) and Linear Sweep Voltammetry (LSV, scan rate: HER, 1 mV s^−1^; OER, 5 mV s^−1^) measurements were taken electrochemical analyzer, CHI760E, Shanghai) to evaluate the performance of the clusters modified electrodes for HER and OER. HER was carried out in 0.5 M H_2_SO_4_, and a graphite rod and a saturated calomel electrode were used as the counter electrode and reference electrode, respectively. OER was carried out in 1 M KOH, and a platinum wire and an Ag/AgCl electrode were used as the counter electrode and reference electrode, respectively.

CV (scan rate: 50 mV s^−1^) and LSV (scan rate: 5 mV s^−1^) measurements were taken (electrochemical analyzer, CHI760E, Shanghai) to evaluate the catalytic activities for O_2_ and H_2_O_2_ reduction. A three-electrode cell was adopted for both O_2_ and H_2_O_2_ reduction, a platinum wire and a saturated calomel electrode were used as the counter electrode and reference electrode, respectively. Oxygen reduction reaction (ORR) was carried out in O_2_-saturated in 0.01 M PBS (pH 7.4), and the H_2_O_2_ reduction was performed in the presence of 9.8 mM H_2_O_2_ in N_2_-saturated 0.01 M PBS (pH 7.4).

### In vitro evaluation of the catalytic activities

Cytotoxicity assay. mouse hippocampal neuronal cell line (HT22) (3 × 10^3^), mouse microglia (BV2) (4 × 10^3^), and mouse astrocytes (MA-c) (4 × 10^3^) were seeded in 96-well plates filled in 100 µL medium with 0.01 M PBS (Gibco) at the border overnight. The culture medium was replaced by different doses of Au_25_, Au_24_Cu_1_, or Au_24_Cd_1_ dissolved in the DMEM, and then cells were incubated for another 24 h. Wells were washed with 0.01 M PBS once, and the medium were replaced by fresh culture medium with serum-free DMEM. Cell survival was analyzed using MTT (3-(4,5-dimethyl-2-thiazol)-2,5-diphenyl-2 H tetrazolium bromide, Beyotime) of 5 mg/mL for 2.5 h and detected at optical density (OD) 490 nm.

Cell viability and Immunofluorescence. HT22 was used in all the cellular experiments. Cells were seeded into the 96-well plates and grew in Dulbecco’s modified Eagle’s medium (DMEM) at 37 °C with 5% CO_2_. After being stimulated with 100 µM H_2_O_2_ for 6 h, the culture medium was replaced by Au_25_, Au_24_Cu_1_ and Au_24_Cd_1_ dissolved in the DMEM at different doses, and then cells were incubated for another 24 h. HT22 cells (1 × 10^5^ cells per well) were seeded in 6-well plates for 12 h and stimulated with 100 µM H_2_O_2_ for 6 h before being treated with the clusters (12 ng/µL). Cell survival was determined at the MTT concentration of 5 mg/mL for 2.5 h and detected at optical density (OD) 490 nm. Fluorescent staining was carried out to evaluate the intracellular oxidative stress levels with different probes such as DHE for O_2_^·−^ and DCFH-DA for ROS, and cell images were captured by a fluorescence microscope.

### In vivo treatment and behavioral experiment

TBI models: C57BL/6 mice at 21–23 g were employed to establish TBI models using an electromagnetically CCI injury device (eCCI-6.3, Custom Design & Fabrication, Inc), with an impactor of 5 m/s velocity, 0.61 mm depth, 150 ms duration, and 20º angle of dura mater on the vertical axis. The mice were divided into control, TBI, TBI+Au_25_, TBI+Au_24_Cu_1_, and TBI+ Au_24_Cd_1_ group (n = 15) randomly. All mice were anesthetized with 10 % chloral hydrate (10 mg/kg) and the scalp was cut before placed on the stereotaxic frame. The craniotomy was carried out by drilling the skull in a circle of 2 mm in diameter. The scalp was sewn together carefully, and the clusters were added to the wound of TBI mice at a concentration of 50 mg/kg. The healing process was recorded photographically and the wound remaining was calculated after treatment.

Oxidative stress level: Brain tissues were taken out on days 7 and 14 post-treatment, then homogenized in 0.9% physiological saline and analyzed for SOD, GSH/GSSG, MDA, and H_2_O_2_ using commercially available kits. All testing methods are carried out as per the instructions (Beyotime).

Morris water maze tests: Morris water maze (MWM) was conducted on days 13–17 and 26–30 post-treatment as described in the previously reported literature [12, 61, 62]. Briefly, the water maze was divided into four quadrants, and the platform was set in the center of quadrant I. Before spatial learning, visual discrimination learning was performed to determine whether the vision of mice was normal. In this procedure, each animal performed one trial where the platform was placed above the water to determine whether the vision of mice was normal. Animal with the visual problem would be excluded in the Morris water maze test. Each mouse was put into the pool to be trained and learned to search for the platform under the water in the order of quadrant II, III, IV and I with an inter-trial interval (ITI) of 60 min at almost the same time of each day for five days. The test was carried out without the platform on the fifth day, and each mouse was put into the pool at quadrant II and allowed for 60 s to track them.

### Ex vivo verification

Western Blotting: The total protein in the brain tissue was extracted and the content of IL-1β (1:1000), IL-6 (1:1000) and TNF-α (1:1000) was analyzed. SDS-PAGE electrophoresis was performed before transferring to the membrane. Immune responses of the specific antibodies were carried out and the images were captured with autoradiography. All antibodies were purchased from Abcam.

ELISA analysis: Inflammatory cytokines including IL-6, IL-1β and TNF-α were determined by ELISA kits (Abcam, ab100712, ab197742, ab208348, respectively), and the assays were performed as per the instructions provided by the manufacture.

Tissue staining: Brain tissues were taken out at 30 days post-injury and fixed in 4% paraformaldehyde and embedded in paraffin. Immunofluorescent staining was performed with primary antibodies including anti-GFAP, IL-6, IL-1β antibodies as per the instructions of (Abcam). Then the slices were incubated with Alexa Fluor 488/594-conjugated goat secondary antibody for 1-1.5 h at room temperature under dark and counterstained with DAPI. Immunohistochemical staining for IL-6 and mice brain tissue was performed according to the instructions (Proteintech).

Toxicological studies: The biosafety of clusters was measured on male C57BL/6J mice at 7-8 weeks (21-23 g). Mice were treated with 200 µL of clusters at a dose of 50 mg/kg every other day. Hematology and blood biochemical indicators were tested on day 30. The blood sample was obtained from the retroorbital sinus and stored in a test tube containing K2EDTA for testing. The blood sample used for biochemical analysis was allowed to stand for 30 min, and then centrifuged twice at 3500 rpm for 15 min each time, the supernatant was collected and tested. The main organs of the mice were collected and fixed in 4 % paraformaldehyde for 24 h, embedded in paraffin, and mounted on a glass slide (4 μm coronal section). The slices were stained with hematoxylin and eosin to observe the toxicity of clusters in major organs, including the heart, liver, spleen, lung, kidney and testis.

### Statistic methods

Data are presented as mean ± standard deviation (SD) or standard error of the mean (SEM). For multiple comparisons, one-way analysis of variance (ANOVA) was performed using the SPSS 22 software to assess difference in means among groups and compared with the Con and TBI groups, analyzed by ANOVA.

## Supplementary Information

**Additional file 1: Figure S1.** The content of metal elements in the clusters were quantified by ICP-MS. **Figure S2.** X-ray photoelectron spectroscopy of clusters. **Figure S3.** Cell survival of (**a**) HT22, (**b**) BV2 and (**c**) MA-c cells in the presence of various concentrations of clusters. **Figure S4.** Hematology of mice treated with or without gold clusters on day 30. **Figure S5.** Blood biochemistry analysis of mice treated with or without gold clusters on day 30. **Figure S6.** Histology of major organs in mice.

## Data Availability

The datasets used and analyzed during the current study are available from the corresponding author on reasonable request.

## Abbreviations

TBI: Traumatic brain injury
ROS: Reactive oxygen species
OH·: Hydroxyl radical
O_2_^·−^: Superoxide anion radicals
H_2_O_2_: Hydrogen peroxide
RNS: Reactive nitrogen species
NO·: Nitric oxide radicals
ONOO^−^: Peroxynitrite
RONS: Reactive oxygen and nitrogen species
CAT: catalase
SOD: superoxide dismutase
POD: peroxidase
GPx: glutathione peroxidase
HER: hydrogen evolution reaction
OER: oxygen evolution reaction
DLS: dynamic light scattering
GC: glassy carbon
CV: Cyclic Voltammetry
LSV: Linear Sweep Voltammetry
ORR: Oxygen reduction reaction
ICP-MS: Inductively coupled plasma massspectrometry
XPS: X-ray photoelectron spectroscopy
ALT: Alanine aminotransferase
ALB: Albumin
AST: Aspartate aminotransferase
TP: Total protein
CREA: Creatinine
TB: Total bilirubin
BUN: Blood urea nitrogen
WBC: White blood cells
RBC: Red blood cell
HCT: Hematocrit
MCV: Mean corpuscular volume
HGB: Hemoglobin
PLT: Platelets
MCH: Mean corpuscular hemoglobin
MCHC: Mean corpuscular hemoglobin concentration
