# Supplementary material for: Catalytically active gold clusters with atomic precision for noninvasive early intervention of neurotrauma
Source: J Nanobiotechnology. 2021 Oct 13;19:319. doi: 10.1186/s12951-021-01071-4 (PMC8513369; doi:10.1186/s12951-021-01071-4)
Supplement: Supplementary file 1 — Additional file 1: Figure S1. The content of metal elements in the clusters were quantified by ICP-MS. Figure S2. X-ray photoelectron spectroscopy of clusters. Figure S3. Cell survival of (a) HT22, (b) BV2 and (c) MA-c cells in the presence of various concentrations of clusters. Figure S4. Hematology of mice treated with or without gold clusters on day 30. Figure S5. Blood biochemistry analysis of mice treated with or without gold clusters on day 30. Figure S6. Histology of major organs in mice. [file 12951_2021_1071_MOESM1_ESM.docx]

Catalytically Active Gold Clusters with Atomic Precision for Noninvasive Early Intervention of Neurotrauma

**Fig. S1.** The content of metal elements in the clusters were quantified by ICP-MS. Cu and Cd account for 5% and 3%, respectively, indicating successful single atom doping.


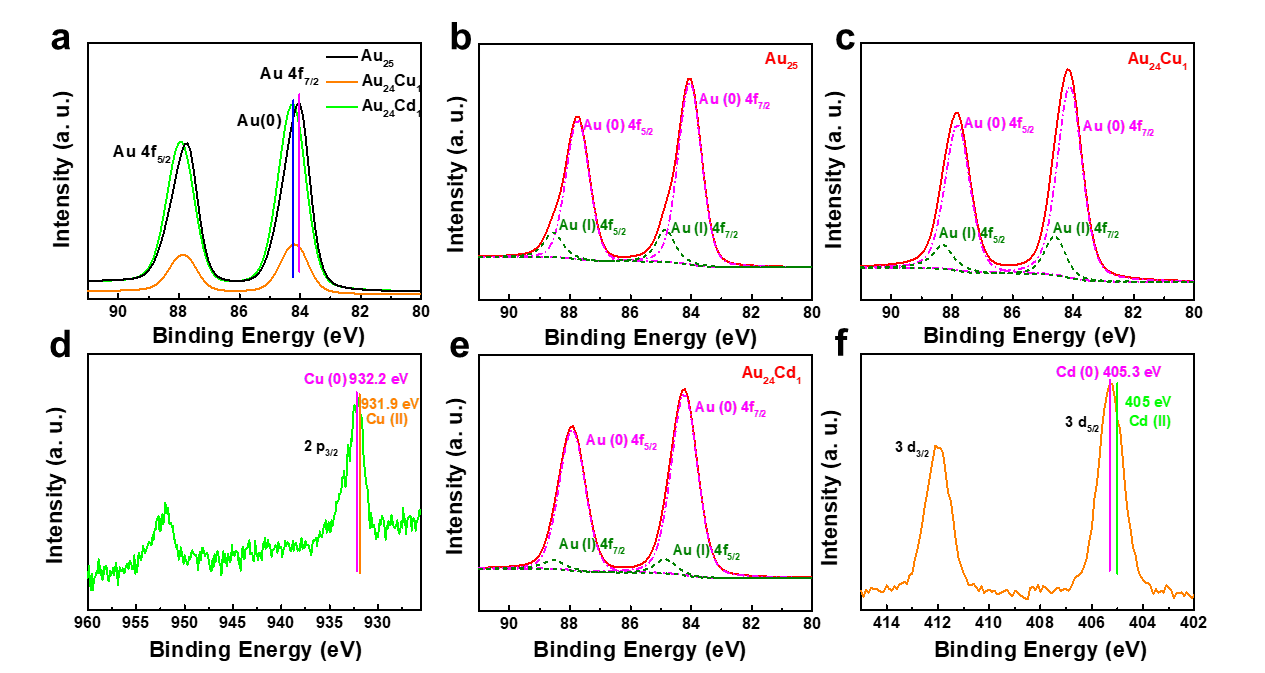


**Fig. S2.** X-ray photoelectron spectroscopy of clusters. (a) Au 4f states of Au_25_, Au_24_Cu_1_ and Au_24_Cd_1_ clusters. High-resolution fine spectra of Au 4f states of (b) Au_25_, (c) Au_24_Cu_1_ and (e) Au_24_Cd_1_ clusters were shown, respectively. (d) Cu 2p XPS spectrum of Au_24_Cu_1_ and (f) Cd 3d spectrum of Au_24_Cd_1_ were shown. All spectra are obtained when the incident energy is 700ev, and all spectra are corrected with 248.8eV C1s peak.


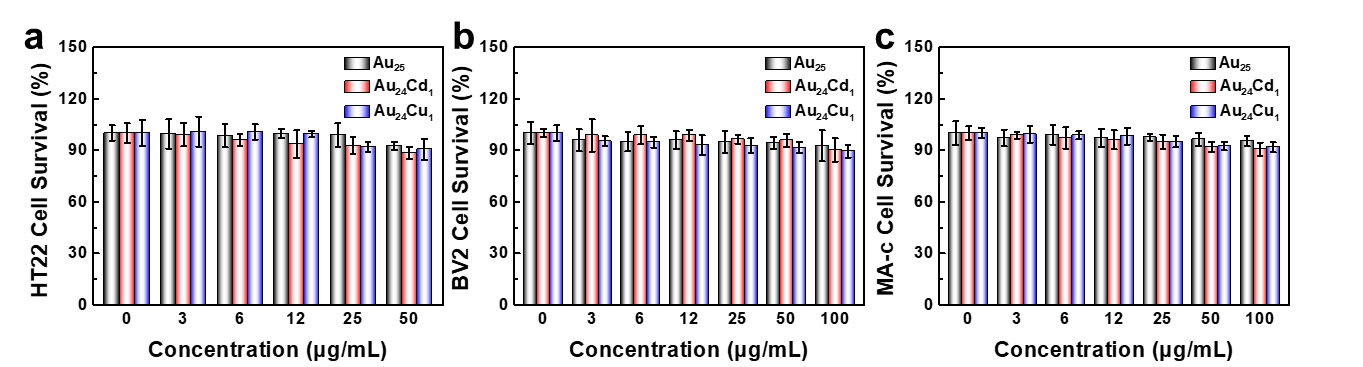


Fig. S3. Cell survival of (a) HT22, (b) BV2 and (c) MA-c cells in the presence of various concentrations of clusters determined by MTT assays (n=5 per group, data are presented as mean ± SD).


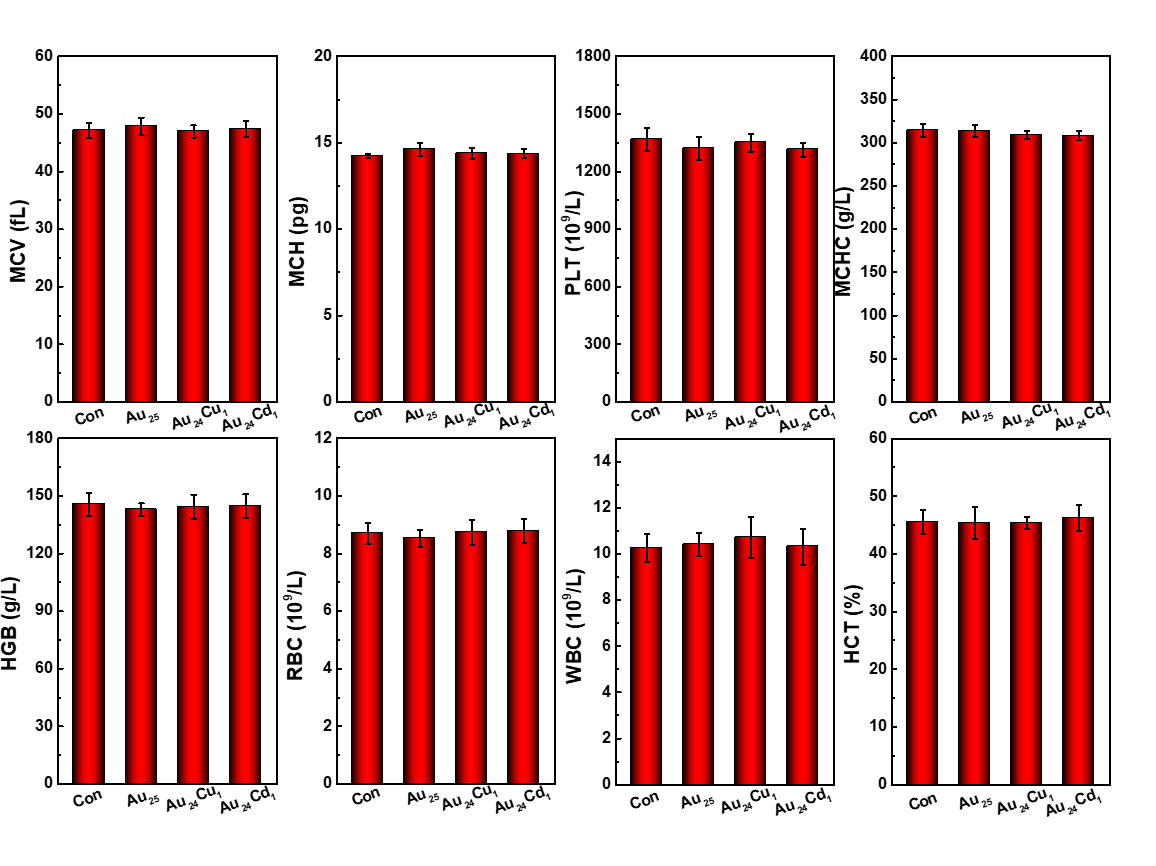


Fig. S4. Hematology of mice treated with or without gold clusters on day 30. White blood cells (WBC), red blood cell (RBC), hematocrit (HCT), mean corpuscular volume (MCV), hemoglobin (HGB), platelets (PLT), mean corpuscular hemoglobin (MCH), mean corpuscular hemoglobin concentration (MCHC) do not reveal any statistical diﬀerences, clearly indicating that no signiﬁcant inﬂammation and infection was induced by the clusters in mice (n=3 per group, data are presented as mean ± SEM).


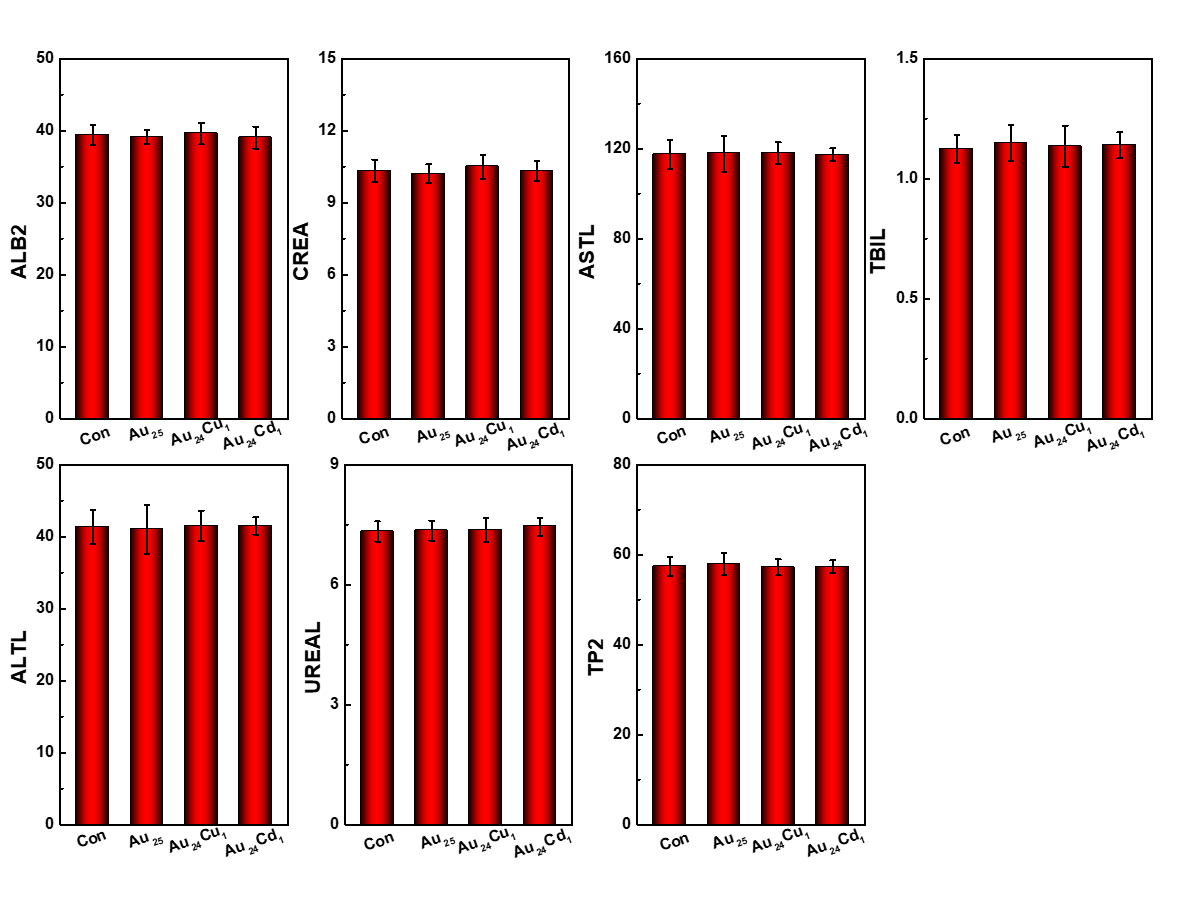


Fig. S5. Blood biochemistry analysis of mice treated with or without gold clusters on day 30. Alanine aminotransferase (ALT), albumin (ALB), aspartate aminotransferase (AST), total protein (TP), creatinine (CREA), total bilirubin (TB), blood urea nitrogen (BUN) levels with a close correlation to the functions of the liver and kidneys of mice do not show statistical diﬀerences between control and treated mice, indicating no signiﬁcant liver and kidney toxicities of clusters (n=3 per group, data are presented as mean ± SEM).


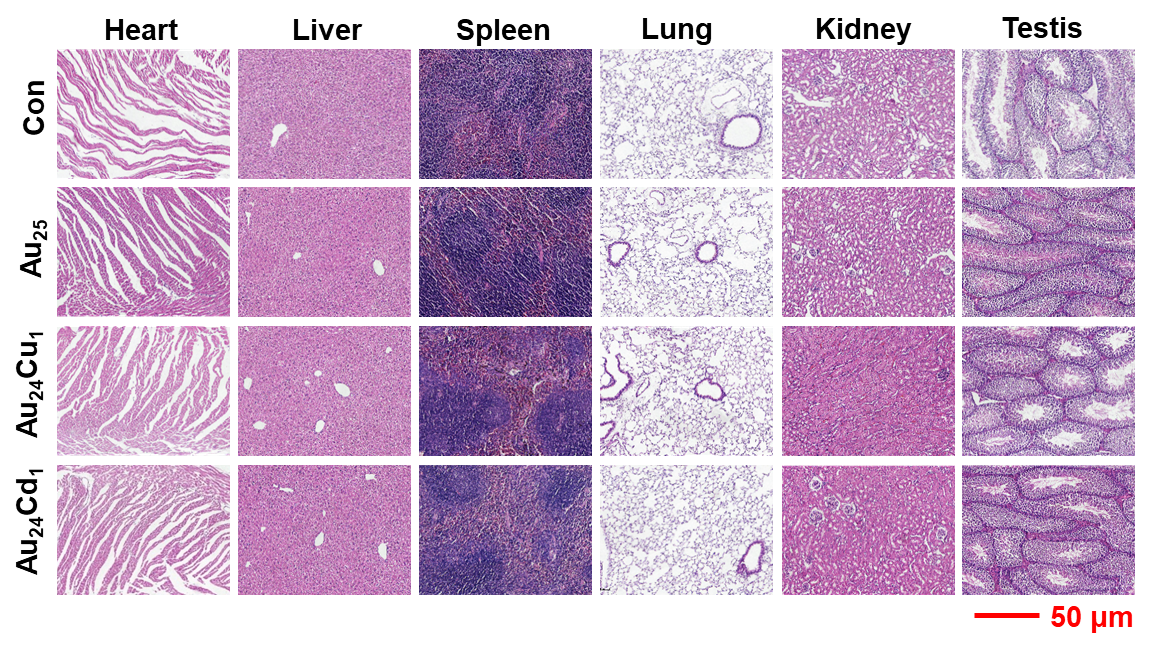


Fig. S6. Histology of major organs in mice (heart, liver, spleen, lung, kidney, and testis) treated with or without gold clusters at the concentration of 50 mg/kg on day 30. Scale bar is 50 μm, n=3 per group. No significant toxic responses were found in all organs.
